# Transcriptional Responses of Olive Flounder (*Paralichthys olivaceus*) to Low Temperature

**DOI:** 10.1371/journal.pone.0108582

**Published:** 2014-10-03

**Authors:** Jinwei Hu, Feng You, Qian Wang, Shenda Weng, Hui Liu, Lijuan Wang, Pei-Jun Zhang, Xungang Tan

**Affiliations:** 1 Key Laboratory of Experimental Marine Biology, Institute of Oceanology, Chinese Academy of Sciences, Qingdao, Shandong, China; 2 University of the Chinese Academy of Sciences, Beijing, China; Chinese Academy of Fishery Sciences, China

## Abstract

The olive flounder (*Paralichthys olivaceus*) is an economically important flatfish in marine aquaculture with a broad thermal tolerance ranging from 14 to 23°C. Cold-tolerant flounder that can survive during the winter season at a temperature of less than 14°C might facilitate the understanding of the mechanisms underlying the response to cold stress. In this study, the transcriptional response of flounder to cold stress (0.7±0.05°C) was characterized using RNA sequencing. Transcriptome sequencing was performed using the Illumina MiSeq platform for the cold-tolerant (CT) group, which survived under the cold stress; the cold-sensitive (CS) group, which could barely survive at the low temperature; and control group, which was not subjected to cold treatment. In all, 29,021 unigenes were generated. Compared with the unigene expression profile of the control group, 410 unigenes were up-regulated and 255 unigenes were down-regulated in the CT group, whereas 593 unigenes were up-regulated and 289 unigenes were down-regulated in the CS group. Gene Ontology and Kyoto Encyclopedia of Genes and Genomes analyses revealed that signal transduction, lipid metabolism, digestive system, and signaling molecules and interaction were the most highly enriched pathways for the genes that were differentially expressed under cold stress. All these pathways could be assigned to the following four biological functions for flounder that can survive under cold stress: signal response to cold stress, cell repair/regeneration, energy production, and cell membrane construction and fluidity.

## Introduction

Environmental stress disrupts homeostasis and can affect biological functions [1]. Temperature has profound effects on the physical and chemical processes within biological systems [2]. Variations in environmental temperature affect many properties and functions of biomolecules and structural components of cells, such as folding, assembly, activity, and stability of proteins [3]; structure and rigidity of lipids [4], [5]; and fluidity and permeability of cell membranes [6], [7].

Rapid decrease in water temperature (e.g., thermocline temperature variation, seiches, and storm events) can lead to many physiological, behavioral, and fitness-related consequences in fish; even small changes in temperature adversely disturb cellular homeostasis and attenuate physiological performance [8], [9]. The adverse effects of temperature fluctuations are overcome and normal cellular functions during altered temperatures are maintained in some fish species via the evolution of versatile mechanisms that enable them to survive in extreme environments [8]. Studies elucidating the mechanisms of temperature acclimation and responses to cold stress in fish have mainly been performed in freshwater fish. Under low temperature conditions, adaptive changes occur in the level of unsaturated fatty acids of cell membrane and ATP is producced by the oxidation of free fatty acids [10]–[12].

The transcriptome is known to be rapidly altered in response to changing environmental conditions. Different techniques have been used to analyze the transcriptional changes in response to temperature in freshwater fish. Microarray analysis revealed a complex adaptive mechanism that promoted cold tolerance in common carp; this mechanism involves the transcriptional regulation of large numbers of genes in different tissues [1]. In zebrafish (*Danio rerio*), many genes that regulate biological processes such as RNA processing, cellular metal ion homeostasis, protein transport spliceosome, proteasome, eukaryotic ribosome biogenesis, and RNA transport were up-regulated by cold stress according to microarray and RNA sequencing (RNA-seq) analyses [14]–[16]. In channel catfish (*Ictalurus punctatus*), cDNA microarray analysis revealed that exposure to cold temperature led to the regulation of genes associated with energy metabolism, angiogenesis, cell stress, muscle contraction and remodeling, and apoptosis [17]. cDNA microarray analysis indicated that annual killifish (*Austrofundulus limnaeus*) exposed to long-term acclimation of different constant temperatures showed changes in the expression of genes associated with cell growth and proliferation, molecular mass chaperones, enzymes in fatty acid saturation, and cholesterol metabolism [18]. Furthermore, microarray analysis suggested that extensive modification of gene expression was needed to maintain the temperature-specific phenotype of heart in rainbow trout (*Oncorhynchus mykiss*) [19].

The olive flounder (*Paralichthys olivaceus*) is native to the subtropical/temperate western Pacific from the Sea of Okhotsk off south-eastern Russia, along Japanese shores, to the South China Sea. Since the 1990s, flounder have been widely cultured in East Asia and are an economically important species in marine aquaculture. Suitable temperatures for flounder range from 14 to 23°C, although they can survive for a short time (a few hours) at near-freezing temperatures (0°C) and even at more than 30°C (findings of our study). Maintenance of temperature, especially in the winter in high northern latitudes, requires installation of heated seawater in many aquaculture farms; this results in a high cost of energy. Cold-tolerant flounder might be a good source to resolve this problem. Cold-tolerant flounder can be selected using molecular marker-based methods [20], [21], and the identification of cold-related pathways and genes might help in not only understanding the mechanism of cold tolerance; but also the artificial selection of cold-tolerant flounder. RNA-seq has been used to identify potential genes or to investigate whole-genome expression in many biological systems for trait selection, including species whose genomic sequences are not yet known [22].

In this study, RNA-seq was used to investigate the differences in transcriptional responses between cold-tolerant and cold-sensitive flounder. The pathways that were mostly activated under cold stress were signal transduction, lipid metabolism, digestive system, and signaling molecules and interaction. Our findings might help better understand the transcriptional basis of cold acclimation among fishes.

## Materials and Methods

### Ethics statement

Experiments on flounder were performed according to the regulations of local and central government. All experiments were approved by the Institutional Animal Care and Use Committee of the Institute of Oceanology, Chinese Academy of Sciences.

### Cold stress

Healthy olive flounder, weighing 25.58±5.36 g and about 5 months of age, were obtained from a commercial supplier (Rizhao, Shandong province) and cultured at 15±0.5°C for 2 weeks in one tank. Flounder were then randomly separated into two tanks. One tank contained 180 flounder that were subjected to cold stress, and the other contained 50 flounder that acted as the control group. For the cold-treatment group, the water temperature was cooled to 0.7±0.05°C at a rate of 1°C/h, and then this temperature was maintained for 18 h. The control flounder were maintained at 15°C.

After cold treatment for 18 h at 0.7±0.05°C, 62 fish survived (could swim freely); these were defined as the cold-tolerant group (CT) group. The remaining fish barely survived (lost their balance and could not swim normally, but had the ability to move); these were defined as the cold-sensitive (CS) group. A total of six individuals per group were anesthetized and immediately sampled. For each fish, three tissues (brain, muscle, and liver) were sampled; and the tissues were stored separately at −80°C until use.

### mRNA-seq library construction for Illumina sequencing

Total RNA was extracted from the tissues by using Trizol Reagent (Invitrogen, CA, USA) according to the manufacturer's instructions. Quality and integrity of RNA were determined using a NanoDrop spectrophotometer (NanoDrop, USA) and Agilent 2100 Bioanalyzer (Agilent, USA). The mRNA was converted to a library for subsequent cluster generation by using Illumina TruSeq RNA Sample Preparation Kit (Illumina, USA). The mRNA was purified using magnetic beads attached to poly-T oligo. Subsequently, the mRNA was fragmented into small pieces by using divalent cations under elevated temperature. First-strand cDNA was generated from the cleaved RNA fragments by using reverse transcriptase and random primers. The second-strand cDNA was synthesized using DNA polymerase I (Fermentas, USA), and RNase H was used to destroy the template RNA. A paired-end library was constructed from the cDNA synthesized using the Genomic Sample Prep Kit (Illumina). After the cDNA fragments were purified using QIAquick PCR (Qiagen, USA) Extraction Kit, they were ligated with adapters as reported previously [23]. The unsuitable fragments were then removed using AMPureXP beads, and a sequencing library was constructed using polymerase chain reaction (PCR) amplification, checked with Pico green staining and fluorospectrophotometry, and quantified using Agilent 2100. The multiplexed DNA libraries were mixed in equal volumes with normalized 10 nM concentration. Three normalized cDNA libraries (the CT group, the CS group, and control) were constructed using RNA from the CT group, the CS group, and control group. Each RNA sample was obtained from three different tissues, and each tissue sample was a mixture of samples obtained from three fish selected randomly from six fish. The library was sequenced using Illumina Miseq platform (Shanghai Personal Biotechnology Co. Ltd, China).

### Illumina sequencing, assembly, and annotation

Transcriptome sequencing was carried out on an Illumina MiSeq platform to generate about 250 bp paired-end raw reads. After the adaptor contamination was removed, the reads were screened from the 3′ to 5′ end to trim the bases with a quality score of Q<20 by using 5-bp windows, and the reads with final length of less than 25 bp were removed. After the adaptor sequences were removed, ambiguous ‘N’ nucleotides (with an ‘N’ ratio of more than 10%) and low quality sequences (with Q<5) were removed, and the quality reads were assembled into contigs, transcripts, and unigenes by using Velvet [24] (http://www.ebi.ac.uk/~zerbino/velvet) and Oases [25] software (http://www.ebi.ac.uk/~zerbino/oases) with default settings except for the K-mer value to obtain contigs and transcripts. All the transcripts were searched against NCBI non-redundant database (ftp://www.ncbi.nlm.nih.gov/) by using the BLAST program (E-value <10^−5^), and the top-hit transcripts were selected as unigenes.

To further annotate the unigenes, the Blast2GO program [26]–[29] was used to obtain gene ontology (GO) annotation on the basis of GO terms related to the non-redundant database annotation by using the BlastX software (http://www.ncbi.nlm.nih.gov/; E-value, <10^−5^). The genes with functional categories were annotated by aligning the unigenes to evolutionary genealogy of genes: Non-supervised Orthologous Groups (eggNOG) database (http://eggnog.embl.de/), a database of orthologous groups of genes. Reads per kilobase of exon per million mapped reads (RPKM) [30] were used to normalize the abundances of transcripts. *P*-values were calculated using DEseq [31]. A two-fold change differential and *p*-value of <0.05 were used to identify the differentially expressed (DE) genes between two groups. The “volcano plot” showed the distribution of DE genes. Significantly, DE genes between the CT group and CS group were determined by comparing the expression patterns of genes between the CT and CS groups while excluding those genes that were not DE between the CT group and control and between the CS group and control. The pathways activated in the CT, CS, and control groups were identified using the Kyoto Encyclopedia of Genes and Genomes (KEGG) database for pathway annotation (http://www.genome.jp/kegg/). The pathways that showed the most differentially expressed genes were identified using KEGG mapper (http://www.genome.jp/kegg/tool/map_pathway2.html).

### Quantitative real-time PCR

Genes identified in the transcriptome sequencing analysis were validated and quantified using quantitative real-time PCR (qRT-PCR). Primers (Table 1) were designed according to Illumina sequencing data by using Primer Premier 5. Total RNA was obtained from the same samples as those used in Illumina sequencing. Reversed cDNA was also synthesized using the PrimeScriptTM RT reagent Kit with gDNA Eraser (Takara). RT-PCR was performed using an Eppendorf Mastercycler ep realplex. β-actin of flounder was used as an internal control to normalize the expression level, and all experiments were performed in six repeat. The reaction was carried out in a total volume of 20 µL, containing 10 µL SYBR Green Master Mix (Takara), 1 µL diluted cDNA mix, 0.6 µL each primer (10 mM), and 7.8 µL RNase-free water. The thermal profile for SYBR Green RT-PCR was 95°C for 20 s, followed by 40 cycles of 95°C for 5 s, 58°C for 30 s, and 72°C for 30 s. Amplification and detection of only one PCR product was confirmed by performing melting curve analysis of the amplification products at the end of each PCR. After the PCR program, the expression level of different genes was analyzed using the comparative CT method (2^−ΔΔCT^ method [32]).

**Table 1:** Genes and specific primers used for real-time polymerase chain reaction.

Gene name	Primer name	Primer sequence (5′-3′)
*β-actin*	β-actin-F β-actin-R	GGAATCCACGAGACCACCTACA CTGCTTGCTGATCCACATCTGC
*trypsinogen 2*	TRY2-F TRY2-R	AGTCCTTTCCTCCCTCCAGGTATCC ACTCTGCTAAGGTCATCCGTCATCC
*chymotrypsinogen 2*	CHTR2-F CHTR2-R	CCGCTACAACGGCTACACCATCAAC TGACAGACCAGAGGACCACCAGAGT
*trypsinogen 1*	TRY1-F TRY1-R	TGATGTGGTGCTCGCCCATACGC CAAGATCGTCGGAGGGTATGAGTGC
*Sterol-14-alpha demethylase*	CYP51-F CYP51-R	TGGCTCACGGTTTTCTCCTCTTG CCGACCGCTACCTCAATGACAAC
*cGMP-specific 3′,5′-cyclic phosphodiesterase 9A*	cGMP-PDE-F cGMP-PDE-R	CAGTTCAGCAGAGCCTTTCGCATC CTCATCTCGTAGCCTCTTCAGGTCG
*trypsinogen 3*	TRY3-F TRY3-R	CACGGATGACAGGATTGTAGGCG GTCCAGGGTGGTGTAGTCGTAACTC
*elastase4*	LA4-F LA4-R	CGCTGTAGGAACTGACTTGGGTG TGGGGCGTTCACGGTATTGTC
*epidermal growth factor receptor kinase substrate 8*	EGFRK8-F EGFRK8-R	AAGTCACCTCCTGGTTCTATGGG GAGAACAGTTGGGCTCCAGTCAG

## Results

### Transcriptome sequencing and assembly

**Figure 1:**
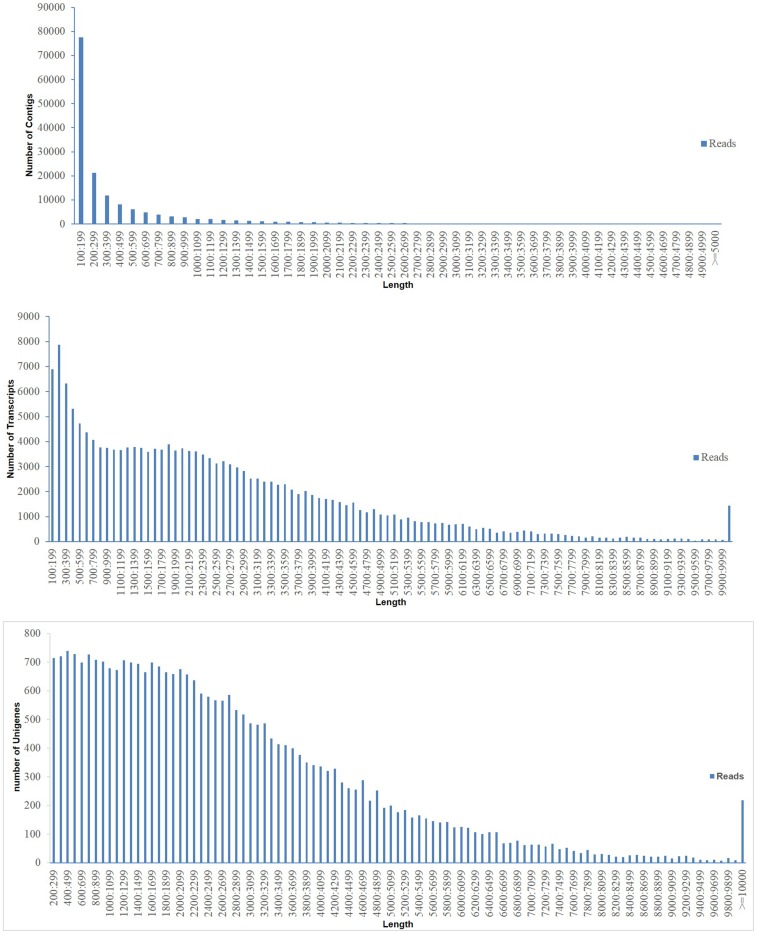
Length distributions of flounder (*Paralichthys olivaceus*) contigs, triscripts and unigenes. Assembly of clean reads resulted in 29021 unigenes that ranged from 201 bp to 57588 bp with a N50 length of 3783 bp. X-axis, length of unigenes; Y-axis, number of unigenes.

In total, there were 10,839,173, 10,260,286 and 8,985,425 raw reads obtained from the control, CT, and CS groups, respectively (Table S1). The average length of raw reads was 251 nucleotides. After the reads were trimmed and quality checked, the quality reads for control, CT, and CS groups were 9,826,199, 9,358,889 and 8,203,714, respectively (Table S2). The quality reads of the three groups were combined and used to draw the transcriptome information of flounder. Assembly of clean reads resulted in 29,021 unigenes that ranged from 201 bp to 57,588 bp with a N50 length of 3,783 bp (Table S3). The length distribution of *P. olivaceus* unigenes is shown in Figure 1. All the sequencing data have been deposited to NCBI under the accession numbers SRX690527.

### eggNOG group analysis

**Figure 2:**
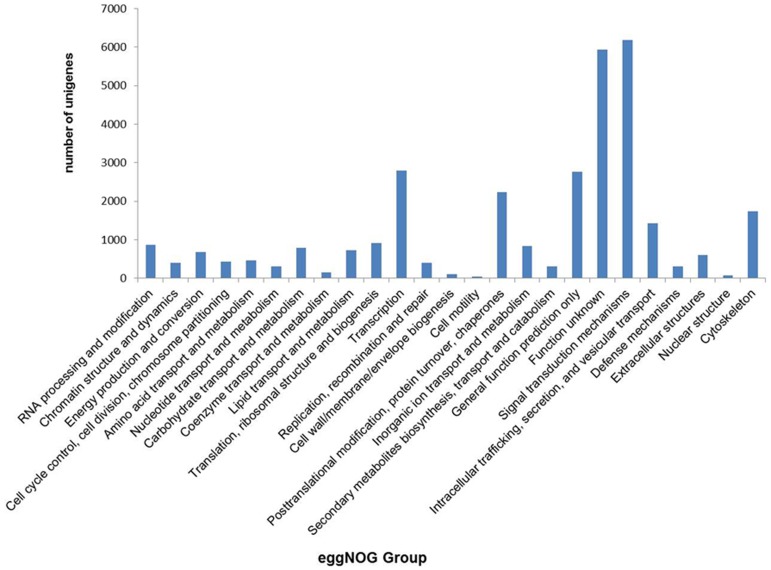
eggNOG group analysis. All 29021 unigenes were analyzed using eggNOG (evolutionary genealogy of genes: Non-supervised Orthologous Groups) database and subdivided into 25 clusters of orthologous (COG) classifications. X-axis, COG classifications; Y-axis, number of unigenes.

Due to the lack of information on flounder total genes, all the unigenes were analyzed using eggNOG database. A total of 29,021 unigenes were subdivided into 25 clusters of orthologous group classifications. Among them, the cluster of “signal transduction mechanisms” (6,181; 19.65%) represented the largest group, followed by “transcription” (2,787; 8.86%), “general function prediction only” (2,761; 8.78%), and “cytoskeleton” (1,738; 5.52%), whereas the cluster of “cell mobility” (45, 0.14%) was the smallest group (Fig. 2).

### Differential expression of unigenes

**Figure 3:**
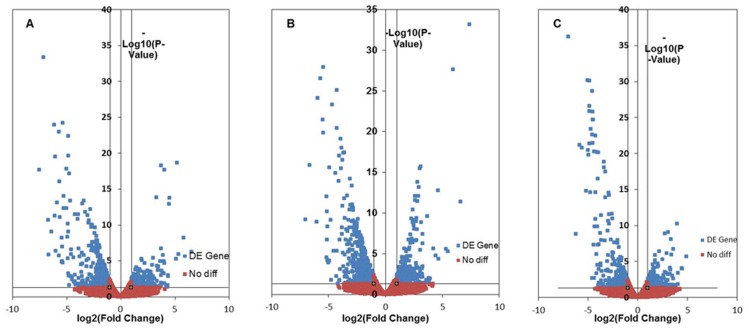
The “volcano plot” picture of differentially expressed genes between two groups. A, the CT group vs. control; B, the CS group vs. control; C, the CT group vs. the CS group. Blue spot, expression fold change of >2 and *p*-value of <0.05; Orange spot, no difference in expression.

Compared with the expression pattern of the control group, there were 410 up-regulated and 255 down-regulated unigenes in the CT group and 593 up-regulated and 289 down-regulated unigenes in the CS group. There were 349 up-regulated and 296 down-regulated unigenes in the CT group compared with those in the CS group (Fig. 3).

### GO classification of DE genes

**Figure 4:**
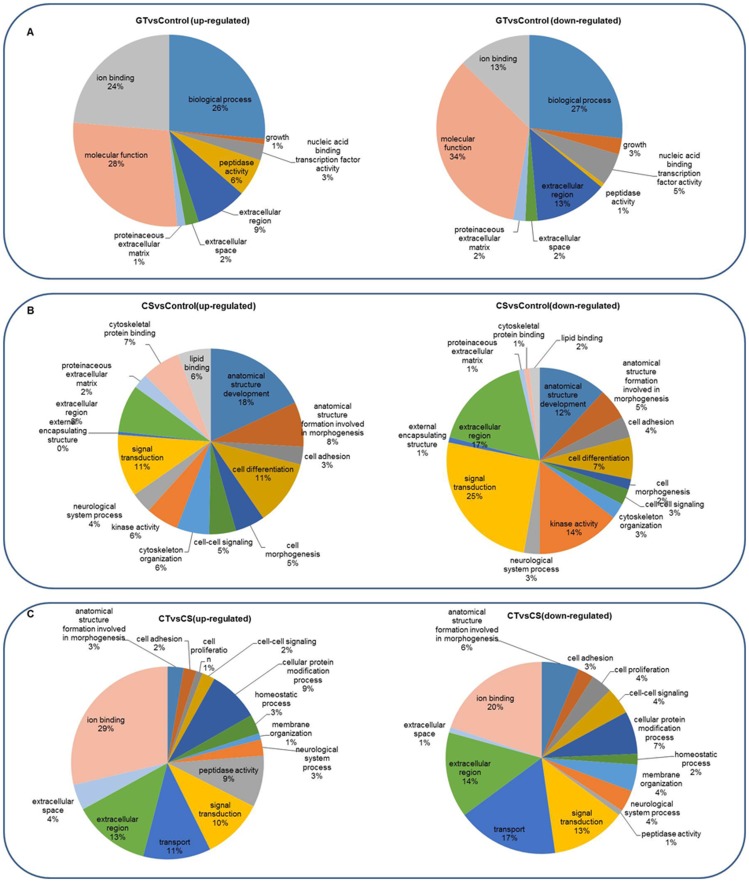
GO classification of DE genes. Annotated DE genes classified into functional categories according to Gene Ontology (GO), Up-regulated and down-regulated genes were classified into two groups. A, the CT group vs. control; B, the CS group vs. control; C, the CT group vs. the CS group.

According to GO, an internationally standardized gene functional classification system, DE unigenes were classified into three major functional categories (biological process, cellular component, and molecular function), which were further divided into 46 subcategories (*p*-value <0.05; Fig. 4).

Comparison with the expression pattern of the control, DE genes of CT group were distributed in three categories and nine subcategories, and the four dominant subcategories were “ion binding,” “extracellular region,” “peptidase activity”, and “nucleic acid binding transcription factor activity.” On the other hand, for the CS group, DE genes were distributed in three categories and 15 subcategories, and the subcategories from dominant to the least dominant were “signal transduction” and “anatomical structure development”; “cell differentiation,” “extracellular region,” “anatomical structure formation involved in morphogenesis”; and “kinase activity.” Comparson of the DE unigenes between the CT and CS groups revealed that nearly one-third of the DE genes were involved in the “ion binding” subcategories, followed by “extracellular region,” “transport signal,” “transduction,” “peptidase activity,” and “cellular protein modification process.”

### Pathway classification enrichment of DE genes

**Figure 5:**
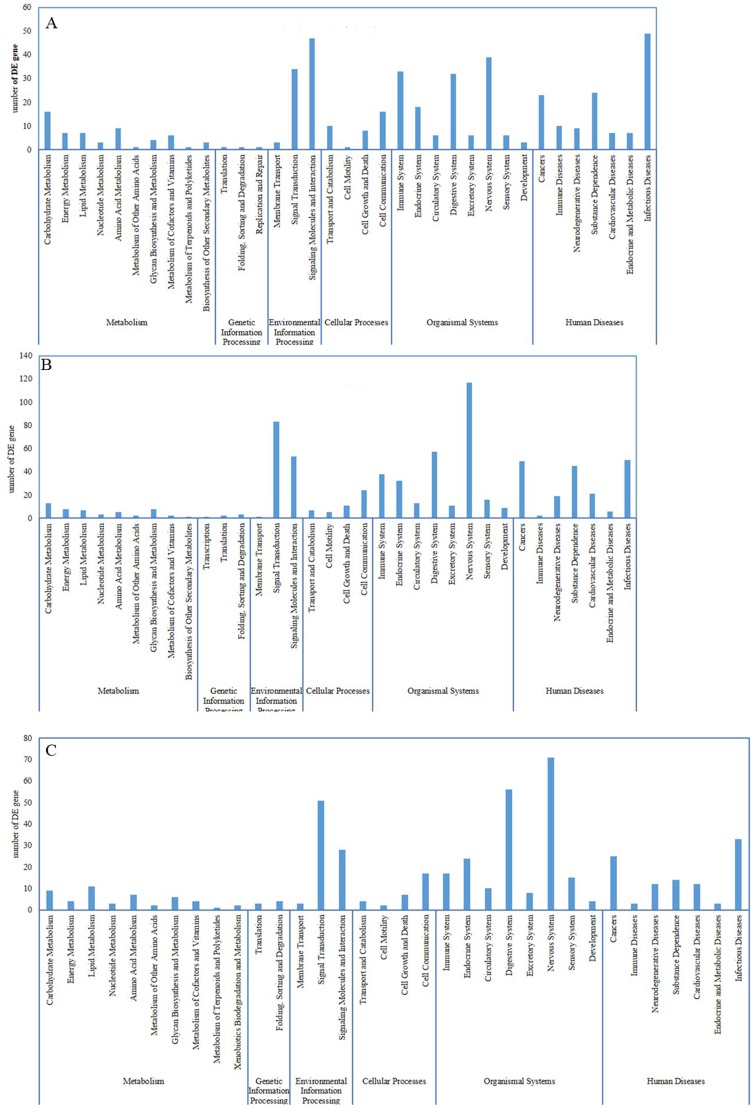
Pathway classification of DE genes. KEGG was used to assign all DE genes to three specific pathways. A, the CT group vs. control; B, the CS group vs. control; C, the CT group vs. the CS group.

KEGG was used to align all DE genes to three specific pathways, including environmental information processing, organismal systems, and human diseases (Fig. 5). The DE genes of the CT group vs. control were identified in “membrane transport,” “signaling molecules and interaction,” “digestive system,” “nervous system,” “immune diseases,” “substance dependence,” and “endocrine and metabolic diseases.” The DE genes of the CS group vs. control were identified in “signal transduction,” “signaling molecules and interaction,” “digestive system,” “nervous system,” “sensory system,” “substance dependence,” and “cardiovascular diseases.” The DE genes of CT group vs. CS group were identified in “membrane transport,” “signal transduction,” “signaling molecules and interaction,” “endocrine system,” “circulatory system,” “digestive system,” “nervous system,” and “sensory system.”

### Cold-related gene and pathway analysis

GO and KEGG analyses were performed to determine the temporal transcriptional events that occurred in the DE genes during cold stress. There was an overrepresentation of several GO categories associated with signal transduction, signaling molecules and interaction, membrane transport, and digestive system.

### Signal transduction

RNA-seq revealed that most enriched genes were growth factor ligands (GFR)-mediated signaling pathway, Ras-related GTPase, nitric oxide (NO)–cGMP, and MAPK signaling pathways.

During cold stress, the growth factor (Table S4) such as *EGFR*, *EGF6*, *IGF1*, *FGFR1* and protein kinase, such as *STK17*, *STK2*, *PLC*, and *PKC* genes were up-regulated in both the CT and CS groups. These genes were involved in GFR-mediated signaling pathway.

The genes that were associated with GTPase were regulated by the cold temperature (Table S4). When the expression pattern between the CT and control groups was compared, *ARHGAP9* and *RAP1GAP2* were up-regulated only in the CT group; on the other hand, when the expression pattern between the CS and control groups was compared, *ARHGAP1-X5* and *RASGRF1* were up-regulated only in the CS group. Proteins encoded by these genes participated in the regulation of other genes associated with Ras.

Many genes involved in the MAPK signaling pathway and calcium signaling pathway were affected during cold stress (Table S4). Unlike in the control group, five genes, i.e., *CACNG1*, *CPCK*, *FLNA*, *CDC25B*, and *ARRB*, were up-regulated and *TNFRSF1A* and *PTP* were down-regulated in the CT group, whereas voltage-dependent calcium channel (*CACNA1A*, *CACNA1H*, *CACNA2D4*, *CACNG1*, and *CACNG7*), *CPKC*, *MAP3K12*, *ARRB*, and *FLNA*, were up-regulated; and only the *DUSP* was down-regulated in the CS group. Analysis of the significantly DE genes between the CT and CS groups revealed that three genes (*CACNA1A*, *CACNA2D4*, and *CACNG7*) were down-regulated and *CAMK II* was up-regulated in the CT group.

Two genes associated with the NO–cGMP signaling were significantly DE. One was *sGC*—a sensor of NO—which showed a 101-fold change in the CT group, whereas it showed only a 5-fold change in the CS group. The other was *PDE9A*, the protein encoded by which could hydrolyze the second messenger cGMP [33], [34]; this gene showed a 98-fold change in the CT group, whereas it was not detected in the CS group.

### Signaling molecules and interaction

DE genes associated with signaling molecules and interaction were mainly enriched in the neuroactive ligand-receptor interaction pathway (Table S4). Seventeen genes (such as *ADRA2A*, *HTR1*, etc.) were down-regulated in both the CT and CS groups. The *GABRA*, *GABRB*, and *GABRD* were up-regulated in both the CS and the CT groups. Analysis of the significantly DE genes between the CT and the CS groups shown that *mGluR5*, *LAMA*α5, and *ITG α9* were up-regulated and *GABRB*, *GABRD* and *THR* were down-regulated in the CT group.

### Digestive system

**Figure 6:**
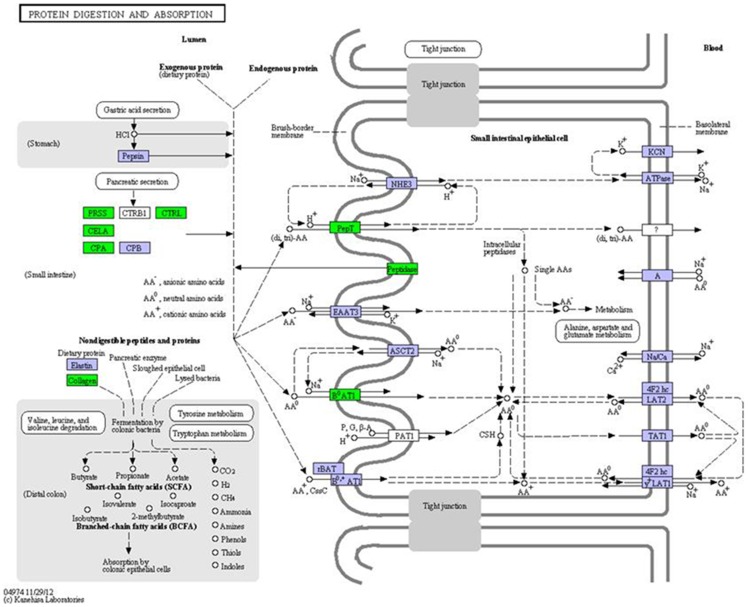
Cold-induced genes associated with protein digestion and absorption pathway. Gene expression value was mapped to the reference pathway by using the KeggArray. Green box,DE genes.

Genes that were involved in the pancreatic secretion pathway were significantly DE (Table S4). Unlike in the control groups, the enzyme-encoding genes (*PRSS*, *CTRL*, *CELA2*, *CPA1*, *CPA2*, *ACEH*, *DPP4*, and *XPNPEP2*), solute carrier family genes (*SLC15A1* and *SLC6A19*), and trypsinogen (*Trypsinogen1, Trypsinogen2*) were up-regulated in the CT group (Fig. 6), whereas all these genes except for *ACEH*, *DPP4*, *XPNPEP2*, and *SLC6A19* were down-regulated in the CS group. In addition, trypsinogen (*Trypsinogen1, Trypsinogen2*) was found to be significantly up-regulated (>10-fold change) in the CT group.

### Membrane transport

In our study (Table S4), the expression of three spliceosomes of ABC transporter proteins was affected by the cold stress: *ABCA1*, *ABCA4*, and *ABCC5*. *ABCA4* and *ABCC5* were up-regulated both in the CT and the CS groups. The change in *ABCC5* expression was significantly higher in the CT group than in the CS group. However, *ABCA1* was down-regulated in the CT group unlike that in the CS group.

### Lipid metabolism

The expression of genes associated with lipid metabolism was also changed by the cold treatment; the DE genes mainly included lipid transport, steroid biosynthesis, and glycerophospholipid biosynthesis (Table S4).

There were six genes associated with apolipoproteins and fatty acid-binding protein (*FABP*), which are involved in lipid transport, that were enriched in the CT and the CS groups. Unlike in the control group, five (*APOAIV1*, *APOAIV4*, *APOD*, *APOE*, and *FABP*) and two (*APOAIV3* and *APOAIV4*) of the six genes were found to be up-regulated in the CT and CS groups, respectively. On the other hand, the expression levels of *APOAIV1*, *APOAIV4*, *APOD*, and *APOE* were significantly higher in the CT group than in the CS group.

**Figure 7:**
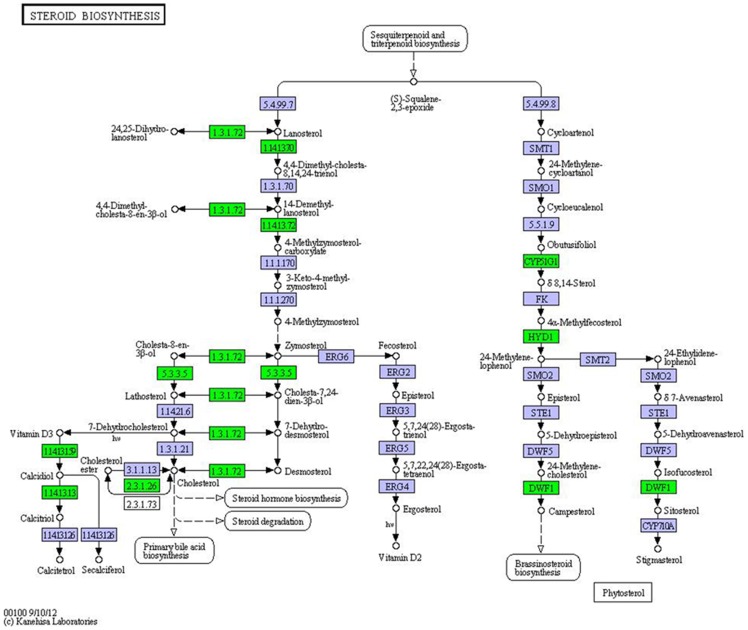
Cold-induced genes associated with the steroid biosynthesis pathway. Differentially expressed (DE) genes were mapped to the reference pathway by using the KeggArray. Green box, DE genes.

Many DE genes involved in steroid biosynthesis and glycerophospholipid synthesis were also DE after the cold treatment. There were seven DE genes (*CYP51*, *DHCR24*, *SOAT*, *CYP2R1*, *CYP27B*, *ERG25*, and *EBP*) associated with steroid biosynthesis pathway (Fig. 7). Unlike in the control group, *CYP51*, *ERG25*, and *EBP* were up-regulated in the CT group, whereas *DHCR24* and *CYP27B* were down-regulated in the CS group. Analysis of significantly DE genes between the CT and the CS groups revealed that *CYP51*, *DHCR24*, *SOAT*, *CYP2R1*, *CYP27B*, *LRP5*, and *PLD* were up-regulated in the CT group; these genes were associated with cholesterol and desmosterol synthesis. After cold treatment, *GPAT*, encoding the enzyme that catalyzes the initial step of glycerolipid synthesis [35], was up-regulated in the CT and CS groups, unlike in the control. However, there was no difference in the expression of this gene between the CT and the CS groups.

### Other cold-related genes and pathways

Many homeobox proteins were also down-regulated in the cold-treated group; these proteins are involved in nucleic acid binding transcription (Table S4). Unlike in the control group, *Hox-A4*, *Hox-A5*, *Hox-B2a*, *Hox-B4a*, *Hox-B8a*, *Hox-C4a*, and *Hox-C5a* were down-regulated in the CT group, whereas *Hox-C6a* and *Hox*-D5a were down-regulated in the CS group. Analysis of the significantly DE genes between the CT and the CS groups showed that the expression of *Hox-A4*, *Hox-A5*, *Hox-B5b*, *Hox-B6b*, *Hox-C5a*, and *Hox-C6a* were down-regulated in the CT group. In addition, *MHC*, associated with muscle fiber phenotype, was only up-regulated in the CT group, unlike that in the control. *ALDO*, involved in glycolysis/gluconeogenesis and pentose phosphate pathway (PPP), was up-regulated in both the CT and CS groups, unlike that in the control. Other representative pathways regulated by cold stress included HIF1, RNA transport, and protein processing. Moreover, pathways associated with cell communication, such as tight junction and gap junction, were overrepresented in the cold-induced genes.

### Validation of RNA-seq data by quantitative real-time PCR

**Figure 8:**
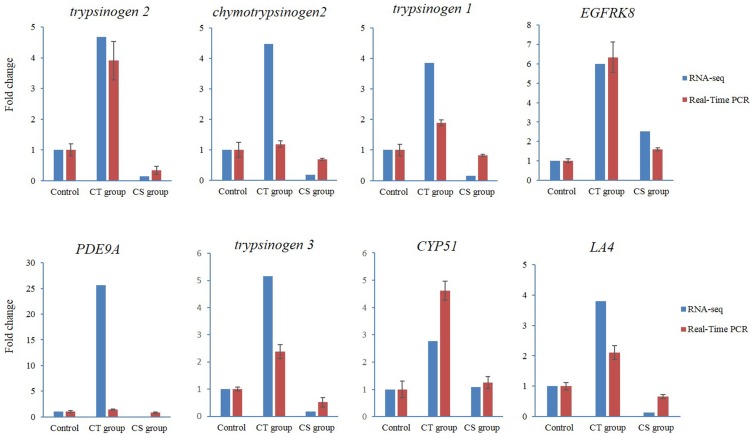
Validation of RNA-seq data by using real-time polymerase chain reaction (PCR). The expression of *trypsinogen2*, *chymotrypsinogen2*, *trypsinogen1*, *CYP51*, *PDE9A*, *trypsinogen3*, *LA4*, *EGFRK8* was detected by RNA-seq (blue column) and qPCR (red column). X-axis, group name; Y-axis, fold change in gene expression.

The expression profiles of genes identified in Illumina sequencing analysis were confirmed by measuring the relative mRNA levels of the following eight genes by using quantitative RT-PCR: *Trypsinogen2*, *Chymotrypsinogen2*, *Trypsinogen1*, *CYP51*, *PDE9A*, *Trypsinogen3*, *LA4*, and *EGFRK8* (Fig. 8). Our results indicated that the data from qRT-PCR were consistent with those of RNA-seq and confirmed the validation of RNA-seq data.

## Discussion

Cold temperature has been an important factor for aquaculture. In this study, RNA-seq was used to compare the differential transcription of genes between the CT, CS, and control olive flounder, marine cultured fish. Numerous genes, key biological processes, and intracellular pathways were involved in cold stress, which might explain the cold tolerance mechanism and can be used to select cold-tolerant flounder. All genes might be assigned to four major groups that might play important roles for facilitating fish survival under low temperature conditions.

### Sensing and intracellular transduction of stress signals

The sensing and intracellular transduction of stress signals is critical for the adaptation and survival of organisms under various environmental stresses [15]. In zebrafish, MAPK signaling were found to be one of the most highly enriched signal transduction pathways under cold tress [15]. In the MAPK signaling pathways, *CACNG1*, *ARRB*, *CPKC*, and *FLNA* were up-regulated in both the CT and CS groups. *CACNG1* is known to be involved in the sensing and transduction of extracellular cold stress signals [36], whereas *ARRB* and *CPKC* are involved in intracellular transduction of stress signals [37], [38]. FLNA could regulate cAMP-dependent pathways by interacting with calcium-sensing receptors [39]. Thus, Ca^2+^ might be an important molecule in the transmission of cold stress signaling from the environment in flounder.

The expression of *CACNA1A*, *CACNA2D4*, and *CACNG7* were significantly lower in the CT group than in the CS group. *CACNA1A* belongs to the calcium signaling pathway. On the other hand, *CaMKII*, which is also involved in calcium signaling pathway, was up-regulated in the CT group unlike in the CS group. *CaMKII* is a multifunctional protein kinase that plays a role in synaptic plasticity, ion channels regulation, and gene transcription [40]; and has been shown to inhibit apoptosis [41]. Interestingly, *CaMKII* inhibitor protein was induced in channel fish brain after cold stress [17]. Further, *mGluR5* and *ITGα9* were up-regulated and *GABR* (*GABRB*, *GABRD*) were down-regulated in the CT group, unlike in the CS group. In the mammalian central nervous system, glutamate is the major excitatory neurotransmitter, and G-protein-coupled metabotropic glutamate (mGlu) receptors can regulate excitability [42]. mGluR5 plays an important role in the central and peripheral nociceptive processes [43], [44]. Integrins, a family of cell surface receptors that attach cells to the matrix, mediate mechanical and chemical signals [45]. *GABR* are a class of receptors that respond to the neurotransmitter gamma-aminobutyric acid (GABA), the chief inhibitory neurotransmitter in the vertebrate central nervous system [46]. The down-regulation of *GABR* (*GABRB*, *GABRD*) might imply that cold stress-related signal transmission was increased. Interestingly, the expression of *SLC6A1* (solute carrier family 6, member 1, also named GABA transporter 1), a neurotransmitter transporter, was up-regulated in zebrafish under cold stress [2].The expression of *PLD*, an important factor for the metabolism of phosphatidylcholine, was also significantly higher in the CT group than in the CS group. PLD can hydrolyze the phosphodiester bond of phosphatidylcholine to produce phosphatidic acid (PA), which might be a secondary messenger and has been implicated in survival pathways that prevent apoptosis [47] and was found to be induced to increase PA in *Arabidopsis* during cold stress [48]. Therefore, the cold signal was transmitted as a positive signal in the CT group. The correct transmission of cold stress signaling from the environment is important for the adaptation and survival or death of flounder under cold stress.

### Cell proliferation/regeneration and/or tissue reparation

Regulation of cell proliferation/regeneration was also important for flounder survival during cold stress. Our results showed that the NO-cGMP and Ras/MAPK signaling pathways were remarkably affected by the low temperature. cGMP is known to be an important signal for cell proliferation [51]. In the CT group, *sGC* and *PDE9A* were up-regulated. NO activated sGC, a sensor of NO, to produce the secondary messenger cGMP whose lifespan could be changed by PDE9A which could hydrolyze cGMP [33], [34]. This link between NO and cGMP was the core of the NO-cGMP signaling pathway [49], which can regulate the cGMP levels [50]. *ABCC5*, another factor that might mediate the NO-cGMP signaling pathway [51] was up-regulated in the CT group. The small guanosine triphosphatase protein Ras is a key mediator of growth factor-dependent cell survival [52], and *RASGRF1*, which was up-regulated in the CS group, is a factor that regulated Ras GTPase activation [53]. Rho GTPase and Rap GTPase, subfamilies of Ras GTPase, have been shown to play roles in the regulation of cell proliferation and apoptosis and can be regulated by their interaction with other effectors such as their associated proteins [54]–[56]. During cold stress, Rho GTPase-associated protein *ARHGAP1-X5* was up-regulated in the CS group, whereas the Rap GTPase-associated protein *RAP1GAP2* and Rho GTPase-associated protein *ARHGAP9* were up-regulated in the CT group. GFR-mediated signaling pathways have been thought to play an important role in cell proliferation [57], and several genes associated with this pathway, such as *EGFR*, *EGF6*, *IGF1*, *FGFR1*, *STK17*, *STK2*, *PLC*, and *PKC*, were up-regulated during cold stress.

During cell proliferation/regeneration or tissue reparation, materials for cell growth should be provided. Apolipoproteins (Apo D, Apo E), which are transporters of cholesterol [58], [59], were only up-regulated in the CT group, and the expression of *LRP5* was also significantly higher in the CT group than in the CS group. These two apolipoproteins have been shown to participate in regeneration within the central and peripheral nervous systems through the reutilization of cholesterol [60]. *APOD* expression might also be related to a differentiation period following proliferation in other cells [60]. LRP5 can bind to ApoE [61] and is required for cholesterol metabolism [62]. During the regeneration of the nervous system, *APOAIV* was found to be up-regulated [63]. In our study, two *APOAIV* (1 and 4) were over-expressed in the CT group, whereas *APOAIV3* and *APOAIV4* were over-expressed in the CS group. This might suggest that different *APOAIV* isoforms might play different roles in cholesterol transport [64]. In zebrafish, *APO* was also up-regulated response to cold stress [2], and Apo E was induced by cold stress in carp [1].On the other hand, another important transporter of cholesterol, *ABCA1* was up-regulated only in the CS group. Over-expressed *ABCA1* increases cholesterol efflux from cells and might be removed by apolipoproteins [65]. Without the cholesterol reutilization process, the cellular cholesterol homeostasis might be disturbed, and this would result in cell death in the CS group. In our study, a key gene *GPAT*, which is involved in glycerophospholipid metabolism, was over-expressed in both of the cold-treated groups. GPAT, the main component of biological membranes, is known to catalyze the initial step of glycerolipid synthesis [35]. The synthesis of glycerophospholipid might compensate for the cell membrane damage caused by cold temperature or for the membranes of new cells developed from regeneration or proliferation. *PLD*, which is also involved in the metabolism of lipids and proteins, was also significantly highly expressed in the CT group than in the CS group. PLD is a lipid anchor and fusogenic lipid and can play a role in the recruitment of proteins to specialized membranes [47]. As the major structural component of all cells in the body, proteins can be synthesized from amino acids or peptides, which are produced from food or body's metabolism. In the CT group, many genes (*PRSS*, *CTRL*, *CELA2*, *CPA1*, *CPA2*, *ACEH*, *DPP4*, *XPNPEP2*, *SLC15A1*, *SLC6A19*, and *Trypsinogen*) involved in protein digestion, absorption, and transportation were up-regulated. On the other hand, in the CS group, some of these genes (*PRSS*, *CTRL*, *CELA2*, *CPA1*, *CPA2*, and *SLC15A1*) were down-regulated. The presence of new cells might suggest that proliferation/regeneration and tissue reparation might occur in the CT group. Therefore, repairing the damaged tissues through regeneration and/or proliferation from quiescent and senescent cells is important for fish to survive during cold stress.

### Energy production

Under cold temperature conditions, the enzymes involved in glycolysis/gluconeogenesis, lipid metabolism, and protein digestion and absorption were enriched, indicating that fish might resist low temperature through energy metabolism. Many genes that are involved in protein digestion and absorption, especially the pancreatic secretion pathway, were up-regulated in the CT group but down-regulated in the CS group (Fig. 6). In our study, flounder were not fed during the cold stress. Without sufficient energy supply from food, proteins might become an alternative source of energy. Under stress conditions, the proteins can served as energy source in fish [13]. In zebrafish, liver protein content was lower in warm acclimated temperature due to their function as energy source [16]. In carp, many genes related to digestive enzyme increased during cold stress [1]. Among these genes, three genes (*PRSS*, *CPB1* and *ELA2A*) were also presented in CT group. ALDO, an important enzyme in glycolysis/gluconeogenesis, was up-regulated, which also was cold-induced gene in zebrafish [2].When flounder were exposed to cold, hypoxia occurred, and the transcription of *HIF-1* was up-regulated. HIF-1 has been shown to increase glycolysis by the up-regulation of glycolytic enzymes [66], [67] and to reduce glucose flux through both the TCA cycle and PPP. *FABP*, a transporter of fatty acids, was up-regulated only in the CT group and also was cold-induced gene in carp and zebrafish [1], [2]. Up-regulated *FABP* might facilitate the metabolism of fatty acids to produce energy by accelerating its transport [68], [69]. Thus, energy was an important factor for flounder to survive in low temperatures.

### Cell membrane adaptive changes

Alteration of the cell membrane might be another important factor that increased cold tolerance. In the CT group, genes involved in steroid biosynthesis (*CYP51*, *DHCR24*, *SOAT*, *CYP2R1*, *CYP27B*, *ERG25*, and *EBP*) were up-regulated. Steroids, such as cholesterol, are used to control the fluidity and flexibility of cell membranes [70], [71]. The permeability, molecular order, elasticity, orientation, and intermolecular spacing of lipid membranes are remarkably dependent on cholesterol content [72]–[75], and high levels of cholesterol could stabilize membranes during cooling [76], [77]. Cholesterol synthesis was shown to be associated with cold tolerance in carp [1]. Cholesterol can also be transported from environment. Only in the CT group, transporters of cholesterol, apolipoproteins (Apo D, Apo E) [58], [59], were up-regulated; further, *LRP5* was significantly over-expressed in the CT group than in the CS group. However, in channel catfish and annual killifish, genes associated with cell membrane respond to low temperature by increasing the level of unsaturated fatty acids [17], [18]. Therefore, the up-regulated genes involved in steroid biosynthesis and cholesterol transport might increase cold tolerance in flounder by stabilizing lipid fluidity and plasma lipoproteins via the enrichment of cholesterol in cell membranes. This suggests that different species use different methods to change cell membrane response to cold stress.

In summary, our study revealed that signal transduction, lipid metabolism, digestive system and signaling molecules and interaction were the most highly enriched pathways for DE genes induced under cold stress in flounder. All these pathways could be assigned to signaling response to cold stress, cell repair/regeneration, energy production, and cell membrane construction and fluidity. Further, we identified some candidate genes that can be used for molecular marker screening, such as (1) genes involved in lipid transport *APO-AIV* family, APO D, *FABP*; (2) genes involved in steroid biosynthesis *CYP51*, *GPAT*; and (3) *sGC* gene, which is involved in the NO–cGMP signaling pathway. The analysis of the regulation and function of these genes under cold tress might facilitate the artificial selection of cold-tolerant flounder that can reduce the energy costs for aquaculture of these species.

## Supporting Information

Table S1 **Raw data of RNA-seq of** ***P.olivaceus*** **.**
(PDF)

Table S2 **Trimmed data of RNA-seq of** ***P.olivaceus*** **.**
(PDF)

Table S3 **Statistical summary of cDNA sequences of** ***P.olivaceus*** **generated by the Illumina Miseq platform.**
(PDF)

Table S4 **Cold-related genes and pathway analysis of** ***P.olivaceus*** **.**
(PDF)
