# Supplementary material for: Transcriptional Responses of Olive Flounder (Paralichthys olivaceus) to Low Temperature
Source: PLoS One. 2014 Oct 3;9(10):e108582. doi: 10.1371/journal.pone.0108582 (PMC4184807; doi:10.1371/journal.pone.0108582)
Supplement: Table S1 — Raw data of RNA-seq of P.olivaceus . (PDF) [file pone.0108582.s001.pdf]

Sample	Reads	Raw Reads	Raw Data(bp)	Reads Len.(bp)
Control	R1	10,839,173	2,720,632,423	251
	R2	10,839,173	2,720,632,423	
	Paired	10,839,173	5,441,264,846	
CS group	R1	8,985,425	2,255,341,675	251
	R2	8,985,425	2,255,341,675	
	Paired	8,985,425	4,510,683,350	
CT group	R1	10,260,286	2,575,331,786	251
	R2	10,260,286	2,575,331,786	
	Paired	10,260,286	5,150,663,572	
