# Supplementary material for: Transcriptional Responses of Olive Flounder (Paralichthys olivaceus) to Low Temperature
Source: PLoS One. 2014 Oct 3;9(10):e108582. doi: 10.1371/journal.pone.0108582 (PMC4184807; doi:10.1371/journal.pone.0108582)
Supplement: Table S2 — Trimmed data of RNA-seq of P.olivaceus . (PDF) [file pone.0108582.s002.pdf]

Sample	Reads	Trimmed Reads	Trimmed Data(bp)	Useful Reads %	Useful Data %
Control	R1	9,826,199	2,239,593,748		
	R2	9,826,199	1,947,704,293	90.65%	76.95%
	Paired	9,826,199	4,187,298,041		
CS group	R1	8,203,714	1,885,857,227		
	R2	8,203,714	1,667,814,646	91.30%	78.78%
	Paired	8,203,714	3,553,671,873		
CT group	R1	9,358,889	2,160,219,161		
	R2	9,358,889	1,881,535,864	91.21%	78.47%
	Paired	9,358,889	4,041,755,025		
