# Supplementary material for: Transcriptional Responses of Olive Flounder (Paralichthys olivaceus) to Low Temperature
Source: PLoS One. 2014 Oct 3;9(10):e108582. doi: 10.1371/journal.pone.0108582 (PMC4184807; doi:10.1371/journal.pone.0108582)
Supplement: Table S3 — Statistical summary of cDNA sequences of P.olivaceus generated by the Illumina Miseq platform. (PDF) [file pone.0108582.s003.pdf]

	Total Length(bp)	Sequence No.	Max Length(bp)	Ave Length(bp)	N50	>N50 Reads No.
contigs	74,165,678	158,416	17,591	468.17	942	20,431
transcripts	444,100,877	174,466	57,588	2,545	3,796	38,914
unigenes	79,656,424	29,021	57,588	2,745	3,783	7,124
