# Supplementary material for: Transcriptional Responses of Olive Flounder (Paralichthys olivaceus) to Low Temperature
Source: PLoS One. 2014 Oct 3;9(10):e108582. doi: 10.1371/journal.pone.0108582 (PMC4184807; doi:10.1371/journal.pone.0108582)
Supplement: Table S4 — Cold-related genes and pathway analysis of P.olivaceus . (PDF) [file pone.0108582.s004.pdf]

category	Pathway	Genes
Signal transduction	growth factor ligands (GFR)-mediated signaling pathway	*Epidermal growth factor receptor* (*EGFR*)<br>*Epidermal growth factor-like protein 6-like* (*EGF6*)<br>*Insulin-like growth factor 1* (*IGF1*)<br>*Fibroblast growth factor receptor* (*FGFR1*)<br>*Serine/threonine-protein kinase 17* (*STK17*)<br>*Serine/threonine-protein kinase 2* (*STK2*),<br>*Phosphatidylinositol phospholipase C* (*PLC*)<br>*Protein kinase C* (*PKC*))
	Ras-related GTPase mediated signaling pathways.	*Rho GTPase-activating protein 1 isoform X5* (*ARHGAP1-X5*)<br>*Ras-specific guanine nucleotide-releasing factor 1* (*RASGRF1*)<br>*rap1 GTPase-activating protein 2* (*RAP1GAP2*)<br>*Rho GTPase-activating protein 9* (*ARHGAP9*)
	MAPK signaling pathways.	*voltage-dependent calcium channel P/Q type alpha-1A(CACNA1A)*<br>*voltage-dependent calcium channel T type alpha-1H(CACNA1H)*<br>*voltage-dependent calcium channel alpha-2/delta-4 (CACNA2D4)*<br>*voltage-dependent calcium channel gamma-1(CACNG1)*<br>*voltage-dependent calcium channel gamma-7(CACNG7)*<br>*classical protein kinase C* (*CPKC*)<br>*mitogen-activated protein kinase* (*MAP3K12*)<br>*beta-arrestin* (*ARRB*)<br>*filamin* (*FLNA*)<br>*dual specificity phosphatase* (*DUSP*)
	NO–cGMP, mediated signaling pathways.	*soluble guanylate cyclase 88E-like* (*sGC*)<br>high affinity *cGMP-specific 3',5'-cyclic phosphodiesterase 9A* (*PDE9A*)

Signaling molecules and interaction		*Adrenoceptor alpha 2A* (*ADRA2A*)<br>*5-hydroxytryptamine receptor 1* (*HTR1*)<br>*Apelin receptor* (*APLNR*)<br>*Proopiomelanocortin* (*POMC*)<br>*Agoutirelated protein* (*AGRP*)<br>*Peptide YY* (*PYY*)<br>*Neurotensin* (*NTS*)<br>*Nociceptin receptor* (*OPRL1*)<br>*Somatostatin* (*SST*)<br>*Tachykinin 1* (*TAC1*)<br>*Arginine vasopressin* (*AVP*)<br>*Oxytocin* (*OXY*)<br>*Neuropeptide FF-amide peptide* (*NPFF*)<br>*Gonadotropin- releasing hormone* (*GNRH*)<br>*Urocortin* (*UCN*)<br>*Adenylate cyclase activating polypeptide* (*ADCYAP*)<br>*Glutamate receptor 1* (*GRM1*)<br>*γ-aminobutyric acid receptor (GABRA, GABRB, and GABRD)*
Digestive system	pancreatic secretion pathway	*Trpsin* (*PRSS*)<br>*Chymotrypsin-like protease*(*CTRL*)<br>*Pancreatic elastase II* (*CELA2*)<br>*Carboxypeptidase A1*(*CPA1*)<br>*Carboxypeptidase A2*(*CPA2*)<br>*Angiotensin-converting enzyme 2* (*ACEH*)<br>*Dipeptidyl-peptidase 4* (*DPP4*)<br>*Xaa-Pro aminopeptidase 2* (*XPNPEP2*)

		Trypsinogen1
		Trypsinogen2
	solute carrier family genes	*solute carrier family 15, member 1* (*SLC15A1*)
		solute carrier family 6, member 19 (*SLC6A19*)
Membrane transport		*ATP-binding cassette, subfamily A, member 1*(*ABC1*)
		ATP-binding cassette, subfamily A, member 4 (*ABCA4*)
		ATP-binding cassette, subfamily C, member 5 (*ABCC5*)
Lipid metabolism	lipid transport	*apolipoprotein A-IV1* (*APOA1*)
		apolipoprotein A-IV3 (*APOA3*)
		apolipoprotein A-IV4 (*APOA4*)
		Apolipoprotein D (*APOD*)
		apolipoprotein E (*APOE*)
		fatty acid-binding protein (*FABP*)
	Steroid biosynthesis and glycerophospholipid biosynthesis.	*Sterol-14-αdemethylase* (*CYP51*)
		Delta24-sterol reductase (*DHCR24*)
		Sterol O-acyltransferase (*SOAT*)
		Vitamin D 25-hydroxylase (*CYP2R1*)
		25-hydroxyvitamin D3 1alpha-hydroxylase (*CYP27B*)
		Methylsterol monooxygenase (*ERG25*)
		Cholestenol delta-isomerase (*EBP*)
		low density lipoprotein receptor-related protein 5 (*LRP5*)
		phospholipase D (*PLD*)
		Glyerol-3-phosphate acyltransferase (*GPAT*)
Other cold-related	nucleic acid binding transcription	*homeobox protein Hox-A4* (*Hox-A4*)
		homeobox protein Hox-A5 (*Hox-A5*)
		homeobox protein Hox-C5a (*Hox-B5a*)

genes and pathway		*homeobox protein Hox-B5b* (*Hox-B5b*)
		homeobox protein Hox-B6b (*Hox-B6b*)
		homeobox protein Hox-C6a (*Hox-C6a*)
	glycolysis/gluconeogenesis and pentose phosphate pathway	*Fructose-bisphosphate aldolase* (*ALDO*)
		myosin heavy chain (MHC)
